# Focal Hepatic Hot Spot (‘Hot Quadrate’) Sign on Contrast‑enhanced Abdominal CT: A Telltale Pearl Indicating Superior Vena Cava (SVC) Syndrome

**DOI:** 10.5334/jbsr.4164

**Published:** 2026-02-10

**Authors:** Maryse Lejoly, Louke Delrue, Koenraad J Mortele

**Affiliations:** 1 Ghent University, Ghent, Belgium

**Keywords:** superior vena cava syndrome

## Abstract

*Teaching point:* Transient, topographically delineated, intense arterial enhancement in liver segment 4, associated with early filling of the dilated veins of Sappey, is a pathognomonic abdominal CT sign of superior vena cava (SVC) syndrome.

## Case History

A 75‑year‑old male, previously treated non‑surgically for cT3N3M0 small cell lung cancer (SCLC) and progressing mediastinal lymphadenopathy, was evaluated with CT for recently developing moderate swelling of the face and mild dilation of venous collaterals on the chest wall.

**Figure 1:**
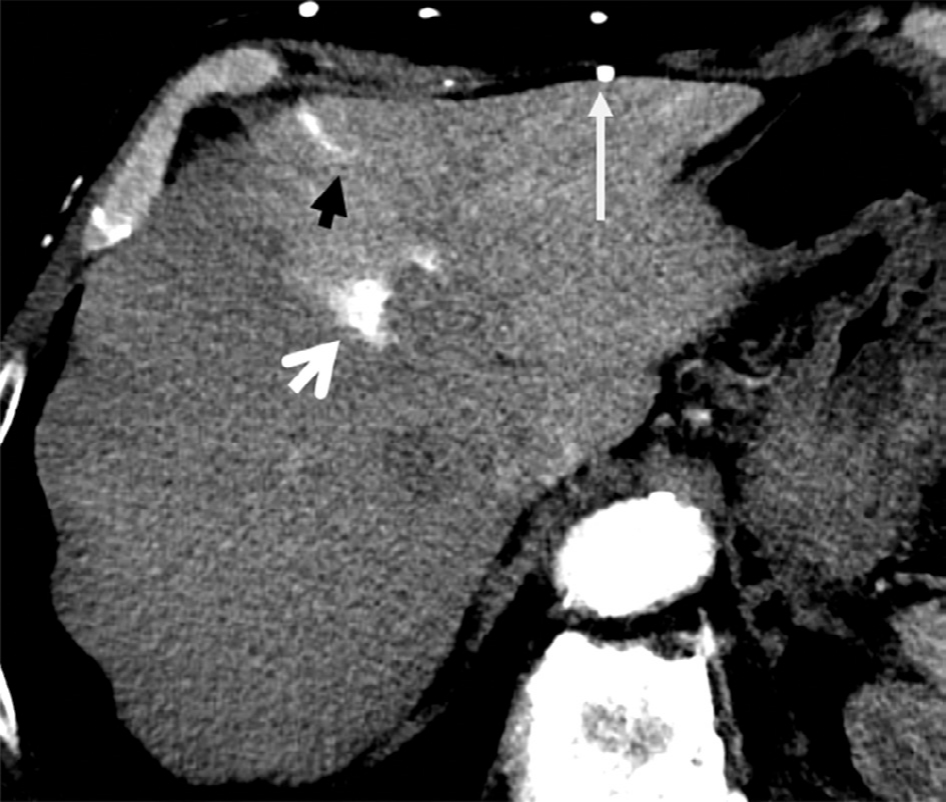
Axial liver CT showing enhancing area in liver segment 4A.

**Figure 2:**
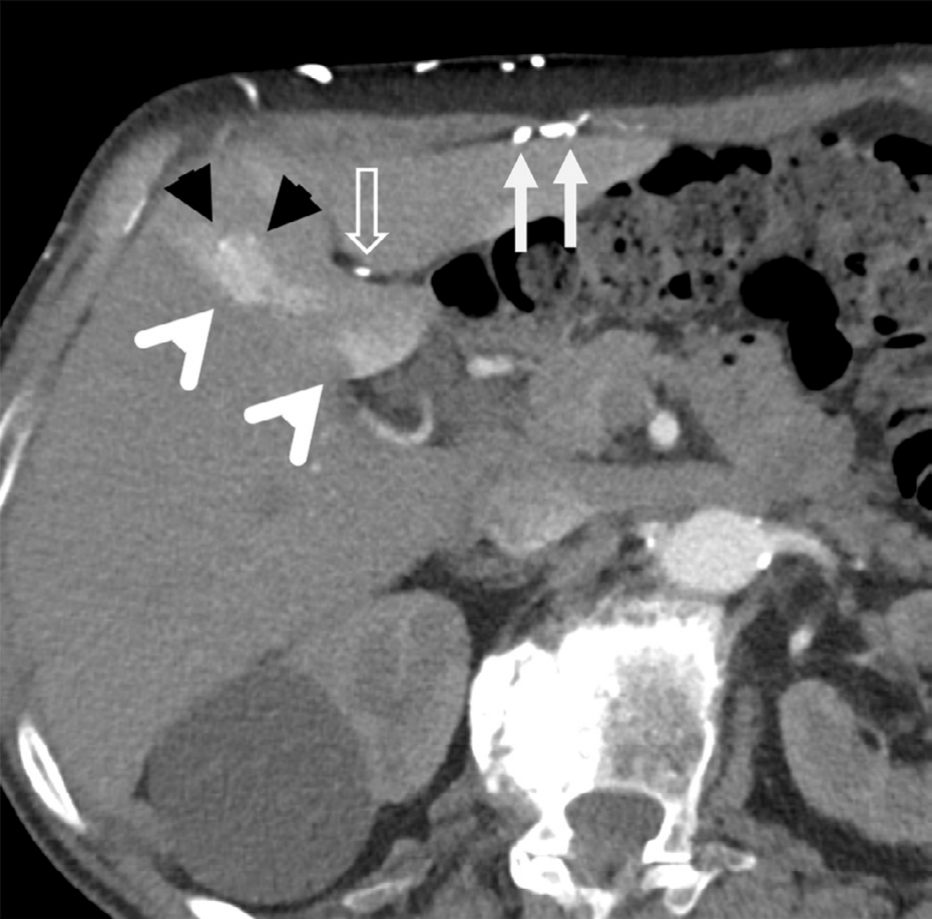
Axial liver CT highlighting segment 4B and collateral vessels.

Arterial phase contrast‑enhanced CT images through the liver showed a triangular intense enhancing area in liver segment 4A, enhancing vessels penetrating into the segment (superior veins of Sappey), and numerous para‑umbilical and subcutaneous collaterals (Figure 1). More inferior, there were also hyper‑enhancing areas in segment 4B, supplied by the inferior veins of Sappey, through para‑umbilical and falciform ligament collaterals, corresponding to the epigastric‑paraumbilical venous system (EPVS) (Figure 2).

**Figure 3:**
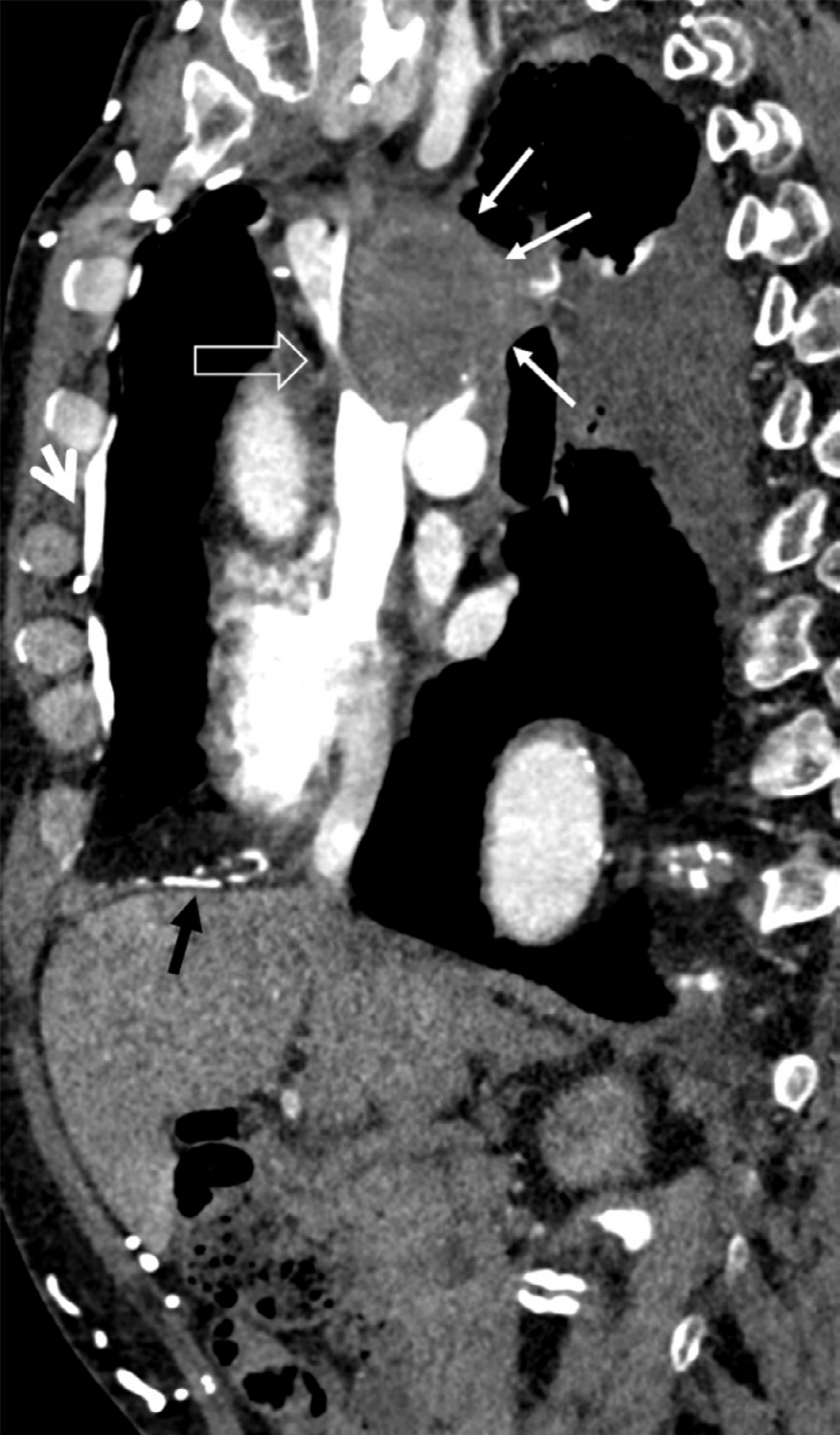
Sagittal CT showing mediastinal mass and SVC occlusion.

Arterial phase, contrast‑enhanced CT of the chest demonstrated a large mediastinal mass with subtotal occlusion of the superior vena cava (SVC) and numerous dilated venous collaterals, including the right‑sided internal thoracic, phrenic, and superior epigastric veins, with branches draining directly into the liver (Figure 3).

## Comments

SVC syndrome is a condition where the SVC is partially or completely blocked, preventing normal blood flow from the head, neck, and arms to the heart. Alike in this case, extrinsic compression and obstruction of the SVC by a malignant neoplasm in the mediastinum is the most common cause. Its onset is mostly insidious, resulting from a rich collateral venous network that diverts blood to the lower body, where it is then returned to the heart through the inferior vena cava, azygos vein, and intercostals [1].

Chest CT with demonstration of collateral vessels is associated with a diagnostic sensitivity of 96% and a specificity of 92%. Contrast‑enhanced CT of the abdomen also typically reveals a pathognomonic observation in liver segment 4, which is known as the ‘focal hepatic hot spot sign.’ Two major collateral pathways are present on CT in SVC syndrome that explain the hepatic findings: (1) one collateral pathway is the SVC–internal thoracic vein (also known as internal mammary vein)–inferior phrenic vein–subcapsular veins (superior veins of Sappey); and (2) another collateral pathway is the SVC–superficial epigastric vein–para‑umbilical/falciform veins–inferior veins of Sappey.

The importance of this sign is that it provides a telltale pearl to the diagnosis of SVC obstruction when enhanced CT of the abdomen is performed in clinically unapparent obstruction. The characteristic location in the quadrate lobe of the liver, the geographically delineated intense enhancement in the arterial phase, in combination with the presence of venous collaterals, are useful clues in differentiating this entity from other focal hypervascular liver lesions.
